# Case-Control Study of Alcohol Usage and Fruit Intake and Stomach Cancer in the North Viet Nam

**DOI:** 10.31557/APJCP.2021.22.9.2903

**Published:** 2021-09

**Authors:** Hoc Hieu Tran, Khanpaseuth Sengngam, Phu Van Pham, Ngoan Tran Le

**Affiliations:** 1 *Department of Surgery, Hanoi Medical University, Hanoi City, Viet Nam. *; 2 *Department of Occupational Health, Hanoi Medical University, Hanoi City, Viet Nam. *; 3 *Department of Nutrition and Food Safety, Hanoi Medical University, Hanoi City, Viet Nam. *; 4 *Institute of Research and Development, Duy Tan University, Da Nang, Viet Nam. *; 5 *Department of Public Health, School of Medicine, International University of Health and Welfare, Japan. *

**Keywords:** Stomach cancer, alcohol usage, fruit intake, case-control study

## Abstract

**Background:**

The aim was to examine the association between alcohol usage, fruit intake and stomach cancer treated in hospitals in the Hanoi city during 2018-2019.

**Methods:**

A case-control study was performed for 379 newly incidence cases of stomach cancer and matched 1096 hospital controls for sex and age (+/-5). We used the validated semi-quantitative food frequency questionnaire to collect data on the intake of alcohol and fruits and other food groups. The average amount of total fruits intake (grams per week) was estimated. The adjusted Odds ratio and 95% confidence interval (OR (95%CI) were estimated.

**Results:**

Intake of alcohol significantly increased the risk of stomach cancer, the mean frequency of intake per year of 345.1 times vs. non-drinkers, OR (95%CI): 1.51 (1.05, 2.17), *p*__trend_=0.026. In contrast, a higher total of fruits intake was associated with a significantly decreased risk of stomach cancer in both sexes, men, and women, (Q5 vs Q1), OR (95%CI): 0.47 (0.30, 0.72), *p*__trend_=0.000, OR (95%CI): 0.45 (0.26, 0.77), *p*__trend _=0.003, OR (95%CI): 0.52 (0.24, 1.12), *p*__trend_=0.026, respectively.

**Conclusions:**

We observed alcohol usage increased the risk of stomach cancers. In contrast, a total of fruits intake was associated with a decreased risk of stomach cancer.

## Introduction

Viet Nam’s population was over 96.2 million on 1 April 2019, national census, ranking 15^th^ most populous country in the world and the third in Southeast Asia. The country is in a sub-tropical region with the main product of agriculture activities and is rich in fruits and vegetables that have been believed to protect against many cancer sites including the stomach. Both fruits and vegetables are rich in micronutrients and contain antioxidants to prevent cancer risks (Chakraborty et al., 2020). Fruits probably protect against stomach cancer (WCRF, 2007). However, only about one third of study participants have a daily intake of fruits and vegetables due to limited knowledge, attitude, and practice of cancer presentation by healthy diet (Falah Asadi et al., 2018). Therefore, more studies on the benefit intake of these organic natural foods are needed. In contrast, the ecological analysis of current by alcohol usage by compared two populations of Japan (high incidence of stomach cancer) and Thailand (low incidence of this cancer) has found that proportion of study participants in Japan (35%) was significantly higher than in Thailand (2.7%) (Pittayanon et al., 2018). 

Among non-communicable diseases occurrences in Viet Nam, cancer was the second leading cause of death in 2012 (WHO, 2014; WHO, 2015). Stomach cancer remained important public health that was the third most common cancer incidence in 2018 with an estimated number of 11,161 (12.3% of 164,671 total new cancer cases) (IARC, 2019). There is a lack of information on the association between environmental factors and stomach cancer in the country. We examined the association between alcohol usage, fruit intake and stomach cancer treated in hospitals in Hanoi city during 2018-2019. 

## Materials and Methods

**Figure 1:**
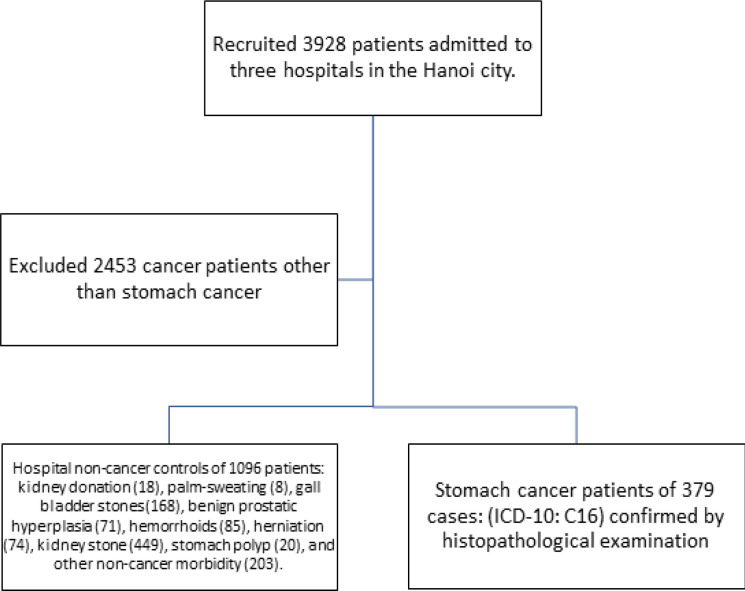
Flow Charge of Study Participants Recruited

A case-control study was conducted for 379 newly incidence cases of stomach cancer and matched 1096 hospital controls for sex and age (+/-5) from three hospitals located in the Hanoi city named Bach Mai, Hanoi Medical University, and National Cancer hospitals, Figure 1. 

*Case recruitment*

We investigated 379 cases diagnosed having stomach cancer (ICD-10: C16) confirmed by histopathological examination during 2018-2019. Cases were weekly selected from the list of all patients admitted to the Bach Mai, Hanoi Medical University, and National Cancer hospitals for surgical treatment to remove stomach cancer tumors for the first time. 

*Control recruitment*

We recruited 1096 patients who have admitted been to the same hospital due to the following diseases: kidney donation (18), palm-sweating (8), gall bladder stones (168), benign prostatic hyperplasia (71), hemorrhoids (85), herniation (74), kidney stone (449), stomach polyp (20), and another non-cancer morbidity (203). They have no history of suffering from any cancer in their lifetime. 

*Exclusion*

Exclusion criteria required for both cancer cases and controls in case they were unable to communicate due to advanced disease stages; patients substantially changing in their diet due to metabolic disorders, and diabetes; and patients refused to participate in the study. 

*Assessment of alcohol usage and fruit intake*

We used the validated semi-quantitative food frequency questionnaire (SQFFQ) to collect the dietary history of participants in the past year from both cases and controls. The SQFFQ has good characteristics of feasibility, practicaly and reliability in general populations. A strong correlation for energy (adjusted R square=0.53), moderate correlation for protein (adjusted R square = 0.38) and carbohydrate (adjusted R square = 0.36) and small correlation for fat intake (adjusted R square =0.20). The SQFFQ included 14 types of fruits and intake of local alcohol products (about 35% or higher alcohol) among 86 questions to collect data on food frequency of intake. Fruits included Guava common; Sugarapple-sweetsop; Orange; Pomelo; Lemon; Papaya ripe; Tangerine-Mandarin; Banana; Banana-dwarf; Watermelon; Litchi-lychee; Longan; Jujube; Apple common. A frequency of intake required seven categories: never or less than 6 times a year, 6-11 times/year, 1-3 times/month, 1-2 times/week, 3-4 times/week, 5-6 times/week, and 1-3 times/day. The SQFFQ is designed for online or offline data collection by a smart device using by the trained interviewers. The interviewers collected data by a face-to-face interview, bedside in hospitals. 

*Data handle and statistical analyses*

The obtained data were exported into both Stata 10 and Excel for double-checking for each record and updated with the final medical records, especially for histopathological confirmation (ICD-10: C16). Body Mass Index (BMI) was calculated as (BMI= weight (kg) / height ((m)^2^) for adjustment.

We estimated the Odds ratio and 95% confidence interval (OR (95%CI) for individual fruit items (higher versus lowest frequency of intake). The adjusted variables included age groups (0-29, 30-39, 40-49, 50-59, 60-69, ≥70 ages), sex, BMI (18.5 to <23, 23 to <25, ≥25, <18.5), an education level (Primary school or under, secondary school, high school, higher high school, unknown), and lifetime smoking (yes/no), intake of total fruits (quintiles), intake of total vegetables (quintiles), intake of total meats (quintiles), and intake of total fishes (quintiles).

*Ethics consideration*

We submitted the research protocol and received the ethic certificate of approval by the Hanoi Medical University IRB for the present study on 25 Dec. 2018. We obtained written informed consent from all participants of the present study.

## Results

The proportion of men was 71.50% of stomach cancer (271 of 379) and 58.58% of controls (642 of 1096). About two-thirds of cases aged 50-69. The proportion of obese (BMI > 25) was 4.49% of cases and 12.59% of controls, Table 1. 

For beverages, there was a null association between a consumption of fresh green tea, drinkers vs non-drinkers, OR (95%CI): 1.07 (0.81, 1.41), p=0.637; both coffee prepared by machine OR (95%CI): 0.88 (0.62, 1.25), p=0.487 or manual filter OR (95%CI): 0.83 (0.52, 1.31), p=0.415, and stomach cancer. Intake of alcohol significantly increased the risk of stomach cancer, the mean frequency of intake per year of 345.1 times vs. non-drinkers, OR (95%CI): 1.51 (1.05, 2.17), *p*__trend_=0.026, Table 2. 

Among 14 types of fruits, there was a null association between fruits intake and stomach cancer for nine types of fruits (Sugarapple-sweetsop; Orange; Pomelo; Papaya ripe; Tangerine-Mandarin; Watermelon; Litchi-lychee; Longan; and Jujube). High consumption of the other five types of fruits (Guava common; Lemon; Banana; Banana-dwarf; and Apple common) was significantly decreased the risk of stomach cancer, Table 3. 

The average amount (grams) of total fruits intake per week, men and women combined, was 177.00 (Q1), 379.30 (Q2), 535.30 (Q3), 703.00 (Q4), and 1485.00 (Q5). A higher average amount of total fruits intake per week was associated with a significantly decreased risk of stomach cancer in both sexes, men, and women, (Q5 vs Q1), OR (95%CI): 0.47 (0.30, 0.72), *p*__trend_=0.000, OR (95%CI): 0.45 (0.26, 0.77), *p*__trend_=0.003, OR (95%CI): 0.52 (0.24, 1.12), *p*__trend_=0.026, respectively, Table 4.

**Table 1:** Characteristics of study participants

Variables	Hospital controls		Stomach cancer	
	n	%	n	%
Sex				
Men	642	58.58	271	71.5
Women	454	41.42	108	28.5
Total	1,096	100.00	379	100.00
Age group				
20-29	37	3.38	2	0.53
30-39	107	9.76	21	5.54
40-49	221	20.16	32	8.44
50-59	315	28.74	122	32.19
60-69	287	26.19	135	35.62
≥70	129	11.77	67	17.68
Total	1,096	100.00	379	100.00
Years of schooling				
<6	155	14.14	66	17.41
6-9	446	40.69	173	45.65
10-12	316	28.83	88	23.22
>12	173	15.78	50	13.19
Unknown	6	0.55	2	0.53
Total	1,096	100.00	379	100.00
BMI (kg/m^2^)				
18.5 - <23	609	55.57	190	50.13
23 - <25	221	20.16	32	8.44
≥25	138	12.59	17	4.49
<18.5	109	9.95	114	30.08
Unknown	19	1.73	26	6.86
Total	1,096	100.00	379	100.00

**Table 2:** Alcohol, Green Tea, and Coffee Intake and Stomach Cancer

Variables	Frequency of intake per year	Hospital controls	Stomach cancer	OR (95%CI) $	P
Local product of alcohol (35% or higher)	1	635	175	1.00 (reference)	
	20.5	254	95	1.27 (0.89, 1.81)	
	345.1	207	109	1.51 (1.05, 2.17)	0.026#
Fresh green tea	1	787	272	1.00 (reference)	
	245.2	309	107	1.07 (0.81, 1.41)	0.637
Coffee prepared by a machine or bags	1	901	322	1.00 (reference)	
	84.9	195	57	0.88 (0.62, 1.25)	0.487
Coffee prepared by a manual filter	1	987	350	1.00 (reference)	
	84.5	109	29	0.83 (0.52, 1.31)	0.415

#p for trend; $Adjusted for age groups (0-29, 30-39, 40-49, 50-59, 60-69, ≥70 ages), sex, BMI (18.5 to <23, 23 to <25, ≥25, <18.5 kg/m2), education level (Primary school or under, secondary school, high school, higher high school, unknown), and lifetime smoking (yes/no), intake of total fruits (quintiles), intake of total vegetables (quintiles), intake of total meats (quintiles), and intake of total fishes (quintiles).

**Table 3:** Frequency of Intake of an Individual Fruit per Year and Stomach Cancer

Fruit name (Vietnamese)	Frequency of intake per year	Hospital controls	Stomach cancer	OR (95%CI) $	*P*__trend_
Guava common (OI)	3.3	211	153	1.00 (reference)	
	24	573	164	0.50 (0.37, 0.67)	
	104	312	62	0.49 (0.33, 0.73)	0.000
Sugarapple, sweetsop (NA)	3.6	389	199	1.00 (reference)	
	24	620	145	0.58 (0.44, 0.76)	
	97.8	87	35	1.63 (0.99, 2.67)	0.271
Orange (CAM)	17	725	232	1.00 (reference)	
	78	296	108	1.47 (0.87, 2.48)	
	262.3	75	39	1.12 (0.41, 3.05)	0.321
Pomelo (BUOI)	16.7	886	310	1.00 (reference)	
	154.8	210	69	1.13 (0.58, 2.20)	-
Lemon (CHANH)	2.5	265	159	1.00 (reference)	
	24	361	113	0.61 (0.45, 0.84)	
	230	470	107	0.55 (0.39, 0.76)	0.000
Papaya ripe (DU DU)	3.7	515	202	1.00 (reference)	
	24	455	153	0.98 (0.74, 1.29)	
	104.9	126	24	0.92 (0.53, 1.58)	0.779
Tangerine (QUIT)	1	189	132	1.00 (reference)	
	20.7	767	196	0.45 (0.33, 0.61)	
	108.1	140	51	1.06 (0.66, 1.69)	0.070
Banana (CHUOI TA)	2.7	233	154	1.00 (reference)	
	24	483	156	0.53 (0.39, 0.71)	
	142.3	380	69	0.38 (0.25, 0.57)	0.000
Banana, dwarf (CHUOI TIEU)	2.5	256	168	1.00 (reference)	
	24	464	142	0.54 (0.40, 0.72)	
	142.8	376	69	0.39 (0.26, 0.59)	0.000
Watermelon (DUA HAU)	3	300	180	1.00 (reference)	
	24	649	157	0.54 (0.40, 0.73)	
	105.1	147	42	1.09 (0.65, 1.84)	0.077
Litchi; lychee (VAI)	3.7	340	190	1.00 (reference)	
	24	679	169	0.66 (0.50, 0.87)	
	107.5	77	20	1.14 (0.62, 2.11)	0.068
Longan (NHAN)	3.8	341	182	1.00 (reference)	
	24	690	169	0.69 (0.52, 0.91)	
	106.3	65	28	2.24 (1.26, 3.98)	0.767
Jujube (TAO TA)	1	262	148	1.00 (reference)	
	19.3	772	204	0.61 (0.46, 0.81)	
	105.1	62	27	1.76 (0.99, 3.13)	0.207
Apple common (TAO TAY)	1	352	209	1.00 (reference)	
	19.4	624	134	0.38 (0.29, 0.51)	
	93.9	120	36	0.84 (0.53, 1.34)	0.000

$Adjusted for age groups (0-29, 30-39, 40-49, 50-59, 60-69, ≥70 ages), sex, BMI (18.5 to <23, 23 to <25, ≥25, <18.5 kg/m^2^), education level (Primary school or under, secondary school, high school, higher high school, unknown), and lifetime smoking (yes/no), intake of total fruits (quintiles), intake of total vegetables (quintiles), intake of total meats (quintiles), and intake of total fishes (quintiles).

**Table 4:** Total Fruit Intake and Stomach Cancer by Sex

Total fruits intake, mean (grams) per week	Hospital controls	Stomach cancer	OR (95%CI) $	*p*__trend_
Both sexes				
177	156	139	1.00 (reference)	
379.3	220	75	0.43 (0.29, 0.62)	
535.3	246	49	0.28 (0.18, 0.42)	
703	235	60	0.39 (0.26, 0.58)	
1485	239	56	0.47 (0.30, 0.72)	0.000
Men				
170	101	97	1.00 (reference)	
378	140	53	0.42 (0.27, 0.65)	
532.9	150	37	0.29 (0.18, 0.47)	
705.3	129	46	0.44 (0.27, 0.72)	
1560.7	122	38	0.45 (0.26, 0.77)	0.003
Women				
191.2	55	42	1.00 (reference)	
381.9	80	22	0.46 (0.23, 0.90)	
539.4	96	12	0.25 (0.12, 0.55)	
699.6	106	14	0.29 (0.14, 0.63)	
1395.2	117	18	0.52 (0.24, 1.12)	0.026

$Adjusted for age groups (0-29, 30-39, 40-49, 50-59, 60-69, ≥70 ages), sex, BMI (18.5 to <23, 23 to <25, ≥25, <18.5 kg/m^2^), education level (Primary school or under, secondary school, high school, higher high school, unknown), and lifetime smoking (yes/no), intake of total fruits (quintiles), intake of total vegetables (quintiles), intake of total meats (quintiles), and intake of total fishes (quintiles).

## Discussion

We observed alcohol usage increased the risk of stomach cancers and in contrast, a total of fruits intake is a strong protective factor against the disease. The findings supported the hypothesis that fruits may decrease the risk of gastric cardia cancers (Vingeliene et al., 2016) and the conclusion of “Fruits probably protect against stomach cancer” by the World Cancer Research Fund/American Institute for Cancer Research (WCRF, 2007). The results were also consistent with the recent conclusions on the inverse association between fruit intake and stomach cancer (Bae and Kim, 2016; Foschi et al., 2010; Gonzalez et al., 2012; Steevens et al., 2011).

Fruits are rich in vitamins, minerals, and other bioactive compounds that may protect against cancers: “Fruits in general probably protect against cancers of the mouth, pharynx, and larynx, and those of the esophagus, lung, and stomach” (WCRF, 2007). 

For alcohol intake, our findings are consistent with some Meta-analyses and an investigation of the mechanisms of alcohol-induced stomach cancer (Han et al., 2017; He et al., 2017; Ma et al., 2017; Na and Lee, 2017; Rota et al., 2017). Alcohol promotes the uptake of carcinogens and their metabolism to induce stomach cancer.

The findings are timely in Viet Nam because the country is located in a sub-tropical region where farm products of fruits are common during four seasons (spring, summer, autumn, and winter) in the North and two seasons (rainy and dry) in the South. With the rapid development of the economy, transportation, refrigerator available at home, people would have more chances to consume daily fruits in general and citrus fruits, in particular, to prevent the occurrence of stomach cancer. To achieve this goal, a program of health education of a healthy diet that in rich in fruits, vegetables, and reduces harmful usage of alcohol is highly recommended and needed. 

The limitation of this case-control study was that the status of *H. Pylori* infection and tumor location in the stomach and types of cancer cells were not available. The spite these limitations, the findings will add new evidence of the harmful usage of alcohol-induced stomach cancer in our study population in a middle-income country. Because the country is located in a sub-tropical region, where farm productions of fruits are common and people have in their hands the means to lead healthy diets, fuller, healthier lives

## Author Contribution Statement

None.
